# Genomic Insights into *Pediococcus pentosaceus* ENM104: A Probiotic with Potential Antimicrobial and Cholesterol-Reducing Properties

**DOI:** 10.3390/antibiotics13090813

**Published:** 2024-08-27

**Authors:** Siriwan Kompramool, Kamonnut Singkhamanan, Rattanaruji Pomwised, Nattarika Chaichana, Sirikan Suwannasin, Monwadee Wonglapsuwan, Jirayu Jitpakdee, Duangporn Kantachote, Thunchanok Yaikhan, Komwit Surachat

**Affiliations:** 1 Department of Biomedical Sciences and Biomedical Engineering, Faculty of Medicine, Prince of Songkla University, Hat Yai, Songkhla 90110, Thailand; siriwantal13@gmail.com (S.K.); skamonnu@medicine.psu.ac.th (K.S.); naampueng.np@gmail.com (N.C.); sirikan4036@gmail.com (S.S.); ythuncha@medicine.psu.ac.th (T.Y.); 2 Division of Biological Science, Faculty of Science, Prince of Songkla University, Hat Yai, Songkhla 90110, Thailand; rattanaruji.p@psu.ac.th (R.P.); monwadee.wo@psu.ac.th (M.W.); jirayu.jitpakdee@gmail.com (J.J.); duangporn.k@psu.ac.th (D.K.); 3 Translational Medicine Research Center, Faculty of Medicine, Prince of Songkla University, Hat Yai, Songkhla 90110, Thailand

**Keywords:** whole-genome sequencing, bacteriocin, gamma aminobutyric acid, genomic characterization, antimicrobial activity

## Abstract

*Pediococcus pentosaceus*, which often occurs in fermented foods, is characterized by numerous positive effects on the human health, such as the presence of possible probiotic abilities, the reduction of cholesterol levels, satisfactory antimicrobial activity, and certain therapeutic functions. This study was conducted with the goal of describing the genomic content of *Pediococcus pentosaceus* ENM104, a strain known for its inhibitory effects against pathogenic bacteria and its remarkable probiotic potential, including the induction of significant reductions in cholesterol levels and the production of γ-aminobutyric acid (GABA). The *P. pentosaceus* ENM104 chromosome is circular. The chromosome is 1,734,928 bp with a GC content of 37.2%. *P. pentosaceus* also harbors a circular plasmid, pENM104, that is 71,811 bp with a GC content of 38.1%. Functional annotations identified numerous genes associated with probiotic traits, including those involved in stress adaptation (e.g., heat stress: *htpX*, *dnaK*, and *dnaJ*), bile tolerance (e.g., *ppaC*), vitamin biosynthesis (e.g., *ribU*, *ribZ*, *ribF*, and *btuD*), immunomodulation (e.g., *dltA*, *dltC*, and *dltD*), and bacteriocin production (e.g., *pedA*). Notably, genes responsible for lowering cholesterol levels (bile salt hydrolase, *bsh*) and GABA synthesis (glutamate/GABA antiporter, *gadC*) were also identified. The in vitro assay results using cell-free supernatants of *P. pentosaceus* ENM104 revealed antibacterial activity against carbapenem-resistant bacteria, such as *Pseudomonas aeruginosa*, *Klebsiella pneumoniae*, and *Acinetobacter baumannii*, and the inhibition zone diameter increased progressively over time. This comprehensive study provides valuable insights into the molecular characteristics of *P. pentosaceus* ENM104, emphasizing its potential as a probiotic. Its notable cholesterol-lowering, GABA-producing, and antimicrobial capabilities suggest promising applications in the pharmaceutical and food industries. Future research should focus on further exploring these functional properties and assessing the strain’s efficacy in clinical settings.

## 1. Introduction

*Pediococcus pentosaceus*, belonging to the genus *Pediococcus* within the Lactobacillaceae family, has become a significant research focus because of its distinctive characteristics and versatile applications across various industries [1,2]. As a Gram-positive lactic acid bacteria (LAB), *P. pentosaceus* exhibits a spherical morphology and possesses a robust homofermentative metabolism, rendering it well-suited for thriving in anaerobic conditions [3]. Its prevalence in a wide array of environments, including fermented foods, aquatic animal products, raw materials, plants, and feces, underscores its ecological significance and adaptive capabilities [4]. One of the key attributes that distinguishes *P. pentosaceus* is its ability to produce a spectrum of antimicrobial compounds, particularly bacteriocins. The antimicrobial activity of *P. pentosaceus* has significant implications for food safety and preservation, as it contributes to the inhibition of microbial growth during fermentation and storage, thereby prolonging the freshness of various food products [2].

Previous studies have shown that *P. pentosaceus* offers various benefits, including food preservation and combating pathogenic bacteria [5,6,7]. Pathogens such as *Salmonella*, *Escherichia coli*, and *Listeria* species, often implicated in food spoilage and foodborne illnesses, are effectively inhibited by *P. pentosaceus* [8,9,10]. Additionally, research has revealed its anti-inflammatory, anticancer, and antioxidant abilities, positioning it as a potential candidate for novel biological drugs aimed at reducing the presence of toxins in the human body [2]. Specifically, *Pediococcus pentosaceus* ENM104, a strain isolated from fermented foods, was chosen for study due to its demonstrated inhibitory effects against pathogenic bacteria such as *Bacillus cereus*, *Listeria monocytogenes*, and *Streptococcus mutans* [11]. Additionally, ENM104 has shown remarkable probiotic potential, including significant cholesterol reduction across various mediums and enhanced antioxidant activities. This strain also produces γ-aminobutyric acid (GABA) and exhibits tolerance to simulated gastrointestinal conditions, further highlighting its suitability as a functional starter culture for dairy products [12].

Given these promising attributes, the current study aims to use bioinformatic tools to analyze the whole genome of *P. pentosaceus* ENM104 to confirm its safety for use as a probiotic in commercial applications and to identify metabolic genes or pathways correlated with the beneficial characteristics of ENM104. Additionally, by investigating its antimicrobial activity, this study seeks to explore its potential medical applications in combating antibiotic-resistant pathogens, thus advancing its application in the development of functional foods, beverages, and therapeutic agents.

## 2. Results and Discussion

### 2.1. Antibacterial Activity of Cell-Free Supernatants (CFSs) from Pediococcus pentosaceus ENM104

In the experiment, two types of CFSs were used: crude CFS and pH-adjusted CFS. The antibacterial effects observed from lactic acid bacteria are primarily attributed to bacteriocins and organic acid substances. To specifically investigate the role of bacteriocins, CFSs were adjusted to neutralize the organic acid present. In the first 12 h, the crude CFS (pH 4.2) had a small effect on inhibiting the growth zone of *Pseudomonas aeruginosa* PA01 and carbapenem-resistant *Acinetobacter baumannii* (CRAB)-SK005 by 15.17 ± 0.34 mm and 12.3 3 ± 0.47 mm, respectively. The inhibition zone gradually increased between 24 and 36 h and reached its highest diameter after 48 h of incubation. Moreover, carbapenem-resistant *Klebsiella pneumoniae* (CRKP10) started to show antimicrobial activity of 12.17 ± 0.29 mm in 48 h (Table 1). However, no antibacterial activity was observed in the pH-adjusted CFS. This indicates that the inhibitory effect of ENM104 may result from the production of antibacterial organic acids or bacteriocins that are active under acidic conditions [13]. However, our previous studies on ENM104 have reported that its antimicrobial activity inhibits the growth of various pathogens including *Bacillus cereus* TISTR 687, *Escherichia coli* ATCC 25922, *Listeria monocytogenes* DMST 17303, *Salmonella* Typhi DMST 22842, *Staphylococcus aureus* ATCC 25923, and *Streptococcus mutans* ATCC 25175 [11]. Therefore, the inhibition activity of the CSF against pathogenic bacteria might be narrow.

Pathogenic bacteria have created severe health issues worldwide, leading to significant antibiotic use, which in turn has contributed to the development of antimicrobial-resistant strains. Hence, the discovery of new alternative treatments, especially agents from natural sources, is an important strategy to control bacterial pathogenesis and reduce the probability of antibiotic-resistant bacteria [14]. Numerous studies have reported the inhibitory effect of LAB against human pathogens, especially *Pediococcus* strains [15]. For example, the supernatant derived from *P. inopinatus* K35 isolated from kimchi exhibits antimicrobial activity against MDR *P. aeruginosa* [16]. Another instance involves *P. acidilactici,* which has demonstrated antagonistic activity against extensively drug-resistant (XDR) *A. baumannii* [17]. Also, *P. pentosaceus* HN10 has exhibited potent inhibitory effects against a range of pathogens, including antibiotic-resistant *Vibrio* spp., *E. coli* ATCC 85922, and *S. aureus* ATCC 25023 [7].

### 2.2. Genome and Plasmid Annotation of ENM104

**Figure 1:**
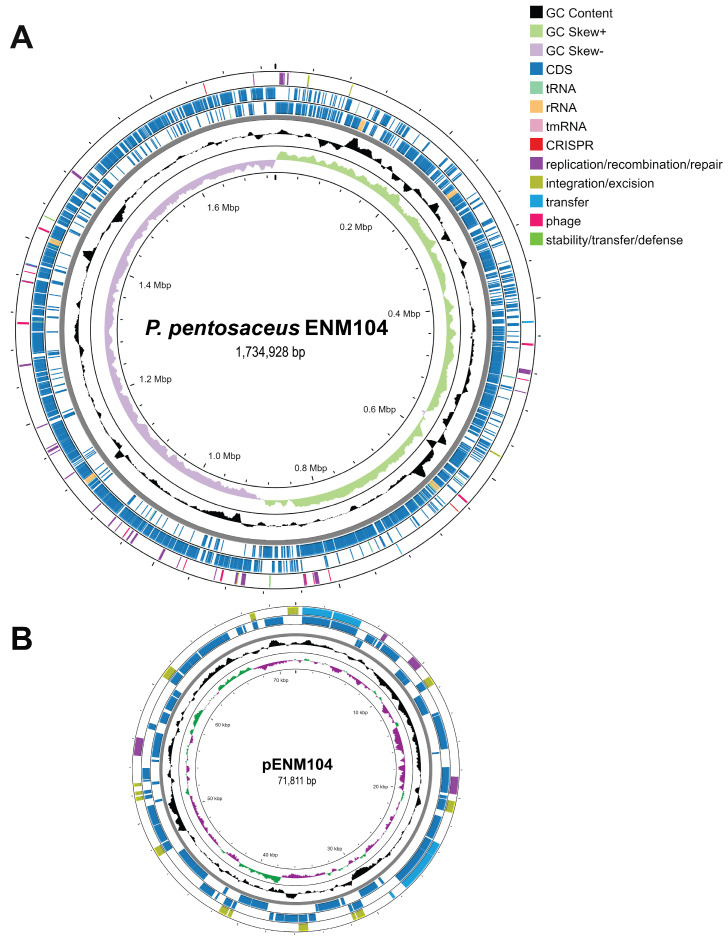
Illustrations of the circular chromosome (**A**) and plasmid (**B**) of *P. pentosaceus* ENM104 present the genomic characteristics in an orderly arrangement. The outermost and second circles depict the positions of the coding sequences (CDSs) in the forward and reverse directions, respectively. The subsequent ring displays additional details, including tRNA, rRNA, GC content, and GC skew (+ and −).

The complete genome sequence of *P. pentosaceus* ENM104 consists of a 1.73 Mbp circular chromosome and a 71,811 bp circular plasmid, named pENM104, as depicted in Figure 1. General information on the ENM104 genome is given in Table 2. Additionally, functional annotations from genome sequences were found for 1689 coding sequences (CDSs) that were assigned to 194 subsystems in the chromosome. Only 417 (25%) out of all CDSs were hypothetical or unknown. Subsystem feature counts of the chromosome are illustrated in Table S1. For plasmid annotations, a total of 85 CDSs were identified in pENM104. Subsystem feature counts of the plasmid are presented in Table S2. Compared with our study, previous research demonstrated that the draft genome sequence of *P. pentosaceus* ST65ACC comprises 1.93 Mbp, with a GC content of 37%. In total, 2012 genes were identified, including 1950 genes with coding sequences (CDSs), 6 genes for rRNA genes, 55 tRNA genes, and 1 tmRNA gene. Interestingly, no plasmids were found in the ST65ACC genome, which differs from the results observed for our ENM104 genome [18]. The genome size of *P. pentosaceus* strains ranges from 1.70 to 2.00 Mbp. This variation is attributed to different environmental selective pressures, which can lead to the retention or loss of specific genes. These genetic changes enable the bacteria to survive and thrive under diverse conditions [19,20,21]. According to the BLASTN result, the pENM104 plasmid was 99.16% similar to the pPP194-1 plasmid from *P. pentosaceus* SRCM100194, suggesting that these plasmids are highly conserved [22]. This suggests that these plasmids are likely to share key genetic elements and functions, which could reflect their adaptation to similar environmental conditions or selective pressures. Moreover, this conservation provides valuable insights into the evolutionary relationships and functional genomics of *P. pentosaceus* strains [23].

### 2.3. Probiotic Properties of P. pentosaceus ENM104

*Pediococcus* strains harbor a multitude of genes responsible for producing proteins that aid in stress responses, allowing them to adjust to conditions within the gastrointestinal tract such as temperature, pH levels, bile salts, osmotic pressure, and oxidative stress. Consequently, the ability to withstand stressful environments is considered a desirable trait in probiotics. In this investigation, we examined the genome of ENM104 to identify various genes associated with probiotic properties, including those related to cell adhesion, resistance to stress, the synthesis of vitamins, immunomodulation, protection, repair of DNA and proteins, biosynthesis of secondary metabolites, and reduction of cholesterol levels (Table 3). Additionally, antibiotic resistance or virulence factor-related genes were not identified in the genome or plasmid of ENM104. This result suggests that ENM104 is a safe strain and can be considered as a probiotic candidate.

**Figure 2:**
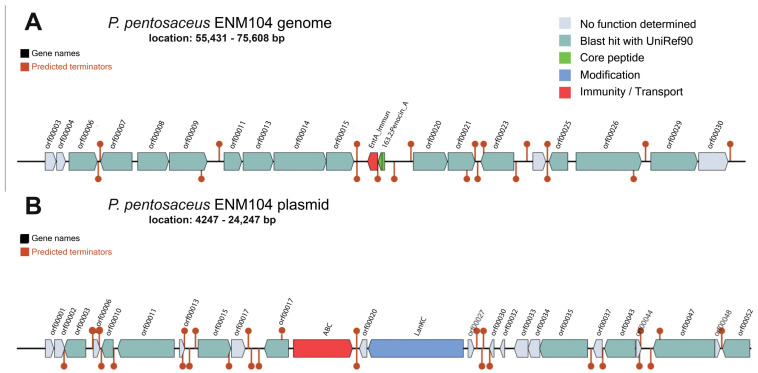
The organization of bacteriocin gene clusters in the *P. pentosaceus* ENM104 genome was predicted using BAGEL4. Notably, penocin_A is located on the chromosome (**A**) and a class IV lanthipeptide is found on the plasmid (**B**).

*P. pentosaceus* has been extensively studied as a probiotic strain and exhibits numerous probiotic effects including antioxidant, cholesterol-reducing, cancer treatment and immune-enhancing properties [24]. Bacteriocin production is also an important characteristic of probiotic bacteria that prevents pathogens, especially foodborne pathogens in the gastrointestinal tract [25]. Interestingly, the BLAST results using BAGEL4 showed that a pediocin-like bacteriocin (*pedA*) termed penocin_A possesses areas of interest (AOI) starting from 55,431 bp and ending at 75,608 bp and was identified in the chromosome of ENM104 (Figure 2A). Pediocin PA-1, a class IIa bacteriocin, has a very narrow spectrum of inhibition directed mainly against the clinically relevant and foodborne pathogen, *Listeria* spp. However, pediocin PA-1 is not active against many other Gram-positive and Gram-negative bacteria [26]. This is consistent with the test results in Section 3.1 for the adjusted CFS. This showed that antibacterial activity was not observed against Gram-negative pathogens. The in vitro study of previous research showed that *P. pentosaceus* ENM104 could inhibit *B. cereus* TISTR 687, *E. coli* ATCC 25922, *L. monocytogenes* DMST 17303, *S.* Typhi DMST 22842, *S. aureus* ATCC 25923, and *S. mutans* ATCC 25175 [11]. The identification of a gene encoding for the bacteriocin penocin_A in ENM104 in this study is consistent with results for other proteins with antimicrobial activity features noted in previous research [11]. Furthermore, *Pediococcus* spp. isolated from raw milk artisanal cheeses have antibacterial activity against various *Listeria* sp. The bacteriocins produced by *P. pentosaceus* strain ST65ACC completely inhibited two *L. monocytogenes* strains including *L. monocytogenes* 211 and 422 [27]. Moreover, a Lanthipeptide_class_IV has an AOI starting from 4247 bp and ending at 24,247 bp, and this gene was found in the pENM104 plasmid of *P. pentosaceus* ENM104 (Figure 2B).

### 2.4. Secondary Metabolite Identification

**Figure 3:**
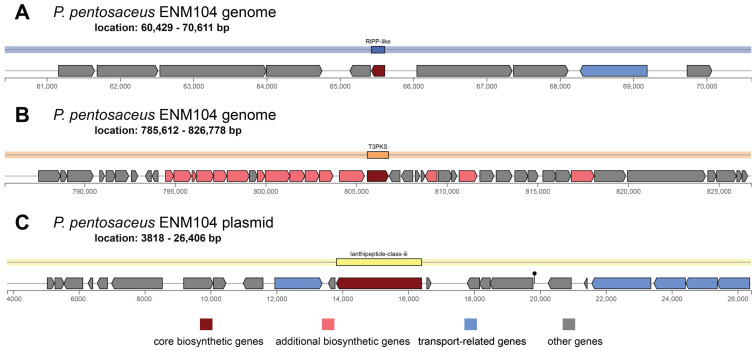
Organization of secondary metabolite clusters in the *P. pentosaceus* ENM104 chromosome and plasmid. The highlighted regions of genes encoding for secondary metabolites found in the plasmid include the following: (**A**) Region 1—RiPP-like cluster, (**B**) Region 2—T3PKS cluster, and (**C**) Region 3—Lanthipeptide class III cluster.

To evaluate secondary metabolite gene regions, antiSMASH was used to predict putative secondary metabolite regions in the ENM104 genome. The BLAST results identified 2 main regions of secondary metabolites. Region 1 is composed of the core biosynthetic gene and the ribosomally synthesized and post-translationally modified peptide (RiPP)-like gene. The RiPP-like gene starts at 60,429 and continues to 70,611 bp (Figure 3A). This RiPP-like region at locus tag ctg1_73, positions 65,429–65,611 bp (total 183 nt), is involved in the biosynthesis of proteins by ribosomes. These proteins then undergo various post-translational modifications to become bioactive such as bacteriocin. The secondary metabolite gene region 2 consists of 7 genes, including type III polyketide synthase (T3PKS) (from 785,612 bp to 826,778 bp) (Figure 3B). T3PKS is a gene associated with the biosynthesis of polyketides, which have been identified as prevalent biosynthetic gene cluster in the LAB strain. A previous study has reported that a polyketide synthase (PKS) system associated with the production of an unidentified secondary metabolite is specifically activated in *Lactococcus lactis* KF147 during growth in plant tissues. Moreover, polyketides are an essential chemical structure that has a wide range of biological activities, including antibiotic, antifungal, anticancer, and immune-modulating properties [28]. It has been proposed that secondary metabolites of polyketides may play a crucial role in the survival of bacteria in the gut environment. Additionally, region 3 was found in the plasmid and is composed of the lanthipeptide class III cluster at position 3818–26,406 bp. This lanthipeptide class III cluster plays an important role in various LAB strains due to their potent antimicrobial activity, which contributes to the preservation of fermented foods and the upkeep of bacterial predominance in intricate environments such as the gut microbiota [29]. Therefore, the results suggest that the ENM104 strain with genes encoding for an RiPP-like gene, T3PKS, and lanthipeptide class III might be utilized as a bacteriocin producer strain with antibacterial action and as a source of natural bioactive products that can have significant bioactive properties and contribute to the beneficial effects in various food and health applications.

### 2.5. Cholesterol-Reducing Gene and GABA Synthesis Gene Identification

The cholesterol-lowering property of probiotic bacteria is an essential feature or survival and colonization in the lower intestine by bacteria with bile salt hydrolase (BSH) activity which plays a role in the enterohepatic cycle. Therefore, BSH activity is considered a crucial factor for colonization [30]. The results revealed that the gene for choloylglycine hydrolase (EC 3.5.1.24), which is involved in bile salt hydrolase activity, was identified in *P. pentosaceus* ENM104. The presence of BSH activity in probiotic strains indicates their potential to lower cholesterol, which is an important indicator in selecting probiotic strains to manage hypercholesterolemia and reduce the blood cholesterol levels in the host. The identification of the gene for choloylglycine hydrolase in ENM104 is consistent with the phenotypic test results reported in a previous study [12]. They found that *P. pentosaceus* ENM104 reduced cholesterol levels by 15.34%, with more significant reductions observed at higher initial cholesterol concentrations due to increased cholesterol degradation. Previous research has shown that probiotics like *Lactobacillus* and *Bifidobacterium* contribute to this effect by enhancing the deconjugation of bile acids, thereby increasing their excretion rates. Cholesterol serves as a precursor to bile acid, and cholesterol is converted into bile acids to replace those lost during excretion, leading to decreased blood cholesterol levels. This process helps regulate serum cholesterol levels by transforming primary bile acids into secondary bile acids using intestinal microorganisms. Additionally, probiotics have the ability to lower cholesterol levels through diverse mechanisms, including absorbing cholesterol during cellular growth, promoting the adherence of cholesterol to cell surfaces, incorporating cholesterol into cell membranes, deconjugating bile acids with BSH, attaching cholesterol to deconjugated bile, binding bile with dietary fiber, and generating short-chain fatty acids from oligosaccharides [31].

Previous studies have indicated that some *Pediococcus* species, especially *P. acidilactici*, contain a gene encoding glutamate decarboxylase [32]. In this study, however, the gene for glutamate decarboxylase (EC 4.1.1.15) was not detected in the ENM104 genome. We identified only the *gadC* gene (glutamate/gamma-aminobutyrate antiporter), which is responsible for importing L-glutamine and exporting either glutamate or GABA [33]. Several *Pediococcus* strains exhibit the capability to produce GABA, although at lower concentrations, which could correlate with our ENM104 strain as shown in Table 4. Despite this, phenotypic analysis of previous study showed that ENM104 produces a small amount of GABA during fermentation, approximately 4.49 ± 0.03 μg/mL [12]. This characteristic aligns with a previous report that *P. acidilactici* LSF1-1 produced approximately 0.80 g/L GABA [34]. Typically, bacteria that produce high concentrations of GABA often possess genes that encode for glutamate decarboxylase alpha (*gadA*) or glutamate decarboxylase beta (*gadB*) in their genome [32]. In our study, the lack of the *gadA*/*gadB* genes may directly explain the lower GABA production observed in these bacterial strains.

In addition to the pan-genome analysis of *P. pentosaceus* strains, *gadA*/*gadB* genes were not detected in any of the strains. However, the *gadC* gene was found in 41 out of 135 strains (30.37%). The absence of *gadA* and *gadB* genes across all examined *P. pentosaceus* strains suggests that these genes are not common in this species, which may explain the generally low levels of GABA production observed. The presence of *gadC* in approximately 30% of the strains indicates that GABA production is not a universal trait among all *P. pentosaceus* strains. This suggests that there might be other pathways or mechanisms responsible for the metabolic activities observed in these bacteria beyond GABA production. The detection of *gadC* in a subset of strains could be linked to specific ecological niches or selective pressures that favor GABA production. Future research should explore the functional role of *gadC* in these strains and investigate the conditions under which GABA production is optimized. This understanding could potentially enhance the probiotic applications of strains that possess *gadC*.

### 2.6. Pan-Genome Analysis

**Figure 4:**
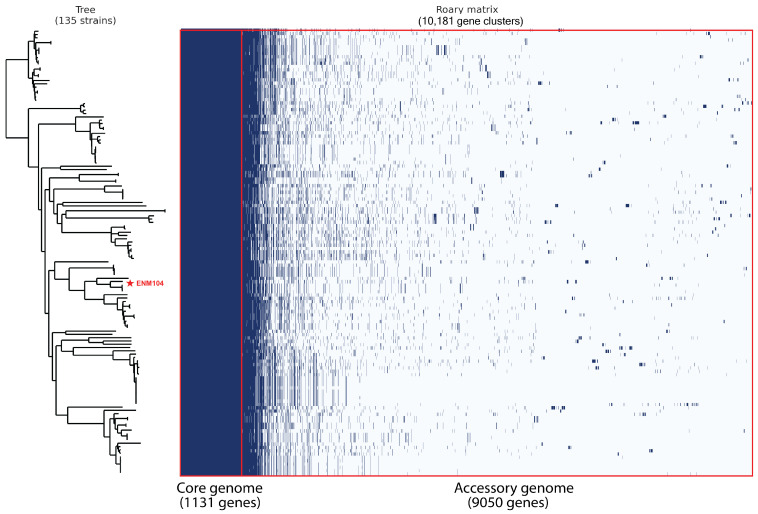
Pan-genome profiles of the ENM104 genome compared with other available *P. pentosaceus* genomes from the RefSeq database from the NCBI. Genes present in each strain are shown in blue, while white indicates the absence of the gene in that species. The red line indicates the boundary between the core genome and the accessory genome.

We conducted a pan-genome analysis to assess the genetic diversity of *P. pentosaceus* strains by comparing the ENM104 strain with other *P. pentosaceus* genomes available in the RefSeq database from NCBI. The analysis included constructing a phylogenetic tree of 136 genomes, including ENM104, based on the SNPs of core genes (Figure 4). We identified 1131 core gene families, which constitute 11% of the total gene families, while the remaining 89% comprised accessory genes. Furthermore, out of 9050 variable genes, 3831 were found to be unique or strain specific. The pan-genome analysis of *P. pentosaceus* reveals significant genetic diversity within the species, highlighting both conserved and variable regions. The core gene families, representing only 11% of the total gene pool, indicate a stable set of essential functions necessary for the survival and basic physiology of the strains. In contrast, most genes are accessory, reflecting the adaptability and specialized functions that different strains may possess.

**Figure 5:**
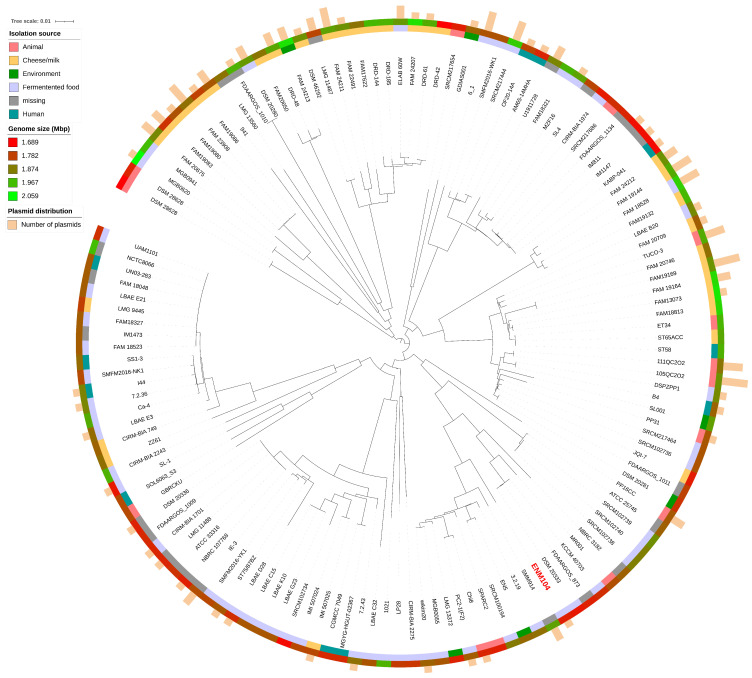
Phylogenetic tree of 135 *P. pentosaceus* strains. The colored ring represents the isolation source with the genome size and the number of plasmids noted.

The identification of 3831 unique or strain-specific genes indicates that individual strains of *P. pentosaceus* have evolved distinct genetic traits, likely as adaptations to various environmental niches or specific functional roles, given that these bacteria were isolated from diverse sources with the majority originating from fermented foods (50 strains), followed by cheese or milk (32 strains), animals (14 strains), humans (11 strains), and different environments (6 strains). However, the isolation sources for 22 strains remain unspecified, as shown in Figure 5. These unique genes may encode factors contributing to strain-specific capabilities, including specialized metabolic pathways, resistance mechanisms, or interactions with hosts or other microbes. In addition, the size distribution of *P. pentosaceus* genomes varies from 1.68 Mbp to 2.10 Mbp. This information aligns with the plasmid identification results for their genomes. Our analysis revealed that over 60 strains contained at least one plasmid marker. Notably, *P. pentosaceus* SRCM100892 (NZ_CP021474.1) harbored seven plasmids, the highest number observed within this species, with sizes ranging from 8 kbp to 55 kbp. The presence of plasmids in *P. pentosaceus* strains also correlates with their adaptive strategies and survival mechanisms [38]. Plasmids often carry genes that confer advantageous traits, such as antibiotic resistance, virulence factors, and metabolic capabilities, which can be crucial for survival in various environments and directly reflect the genome size distribution among this species [23]. This diversity in plasmid size and content can contribute to the strain-specific capabilities observed in *P. pentosaceus*, further explaining the genetic variability highlighted by the presence of unique or strain-specific genes.

In addition to the core genome analysis, the distribution of COG categories among the 1131 core genes of *P. pentosaceus* revealed significant enrichment in several functional categories. The majority of these core genes were associated with metabolism (36.9%), followed by information storage and processing (25.3%), cellular processing and signaling (19.3%), and poorly characterized functions (18.5%). These findings emphasize the metabolic adaptability of *P. pentosaceus*, which is essential for its survival and effectiveness in various environments, such as fermented foods and the gastrointestinal tract. The considerable number of genes involved in information storage and processing highlights the critical role of genetic and regulatory mechanisms in sustaining cellular functions and adaptability. The notable presence of genes related to cellular processing and signaling indicates sophisticated systems for environmental sensing and response, which are crucial for the bacterium’s probiotic functions. This genetic versatility and adaptability make *P. pentosaceus* an invaluable asset in the food industry, particularly in enhancing the nutritional and health benefits of fermented food products.

### 2.7. Comparative Genomic Analysis

**Figure 6:**
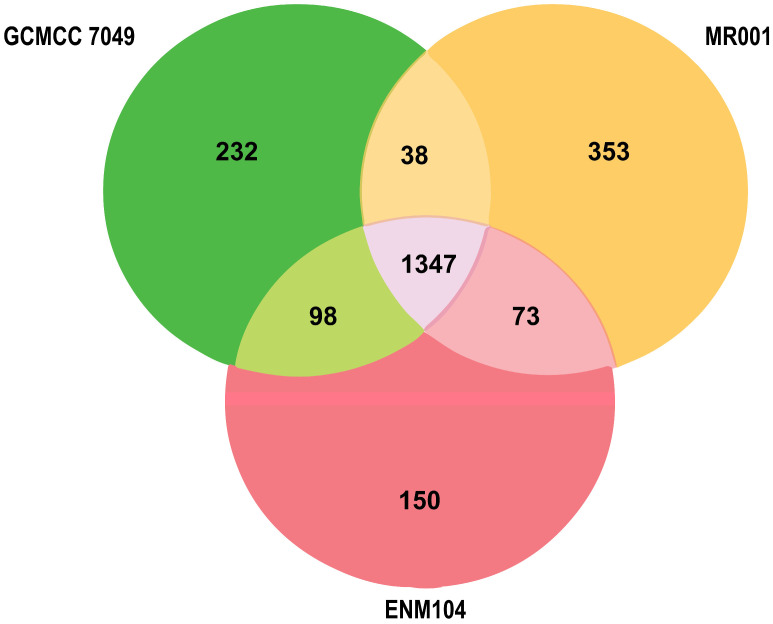
Venn diagrams illustrate the number of reciprocal best matches within the core genomes of *P. pentosaceus* ENM104 (red), MR001 (yellow), and GCMCC 7049 (green) subsets.

Comparative genomic analysis of the ENM104 genome was conducted with its closely related genomes (*P. pentosaceus* MR001 and *P. pentosaceus* CGMCC 7049) based on BLAST identification. A pan-genome overview of our *P. pentosaceus* ENM104 genome compared with two closely related genomes is presented in Figure 6. Comparing the number of annotated genes unique to and shared between the ENM104 genome and those of MR001 and GCMCC 7049 using reciprocal BLAST highlighted distinctive genomic characteristics in ENM104. Additionally, ENM104 exhibited shared core and accessory annotated genes with MR001 (68.97%) and CGMCC 7049 (74.56%). Among the 150 genes identified in the ENM104 genome, two groups were distinguished, encompassing genes related to molecular function and biological processes (Table 5).

In this study, ENM104 carried several unique genes that are involved in various molecular functions and biological processes. Most of these genes were predicted to be hypothetical proteins, while the annotated genes were diverse and present in several microorganisms. For example, *adhR_3* is responsible for HTH-type transcriptional regulator AdhR. This *adhR* is commonly found in other microorganisms, including *B. subtilis*, *E. coli, Clostridium beijerinckii*, and others. These gene families may have been acquired by ENM104 from microorganisms encountered throughout their evolution in various ecological niches [39]. Moreover, AdhR is involved the DNA repair system of *Lactiplantibacillus plantarum* KM1 under H_2_O_2_ stress conditions [40]. Regarding biological processes, the *licT_1* gene is associated with the transcription antiterminator LicT protein that has been reported to regulate the *licS* gene in *B. subtilis*. The *licS* gene participates in the metabolism of β-glucosides and is a member of the BglG/SacY family of antiterminators. It interacts with particular regions in messenger RNAs to inhibit the premature termination of gene transcription by RNA polymerase [41].

## 3. Materials and Methods

### 3.1. Microbiological Characterization

#### 3.1.1. Bacterial Strains

The lactic acid bacterium (LAB) used in this study, *Pediococcus pentosaceus* ENM104 (accession numbers CP137759 and CP137760) isolated from fermented pork sausage, was obtained from a previous study [12]. To grow ENM104 from glycerol stock at −80 °C, the strain was streaked on de Man Rogosa Sharpe (MRS) agar (Himedia, Mumbai, India) and cultured at 37 °C for 24 h followed by culture in MRS broth (Himedia, Mumbai, India) at 37 °C for 24 h. Clinical strains, including *Pseudomonas aeruginosa* PA01, carbapenem-resistant *Escherichia coli* 003 (CREC003), and carbapenem-resistant *Klebsiella pneumoniae* CRKP10, were isolated from patients in the Medicine Ward of Songklanagarind Hospital. These strains exhibit antimicrobial resistance genes (ARGs). Additionally, carbapenem-resistant *Acinetobacter baumannii* strains has been isolated from Songkhla Hospital, Satun Hospital, Pattani Hospital, and Yala Hospital. Characteristics and specimens are listed in Table 6. All clinical strains have been whole genome sequence. All clinical strains were cultured in tryptic soy broth (TSB) (Himedia, Mumbai, India) and incubated at 37 °C with shaking at 150 rpm for 6 h.

#### 3.1.2. Preparation of *P. pentosaceus* Cell-Free Supernatant (CFS)

The overnight culture of *P. pentosaceus* ENM104 was adjusted to the 0.5 McFarland standard and inoculated into MRS broth, followed by incubation at 37 °C with shaking at 250 rpm. Sample cultures were collected at 12, 24, 36, and 48 h. The cell-free supernatant (CFS) from each culture was collected by centrifugation (Centrifuge Sorvall™ ST 16R Refrigerated Centrifuge, Thermo Scientific™, Waltham, MA, USA) at 8000× *g* for 10 min. The pH of the CFS was measured. CFSs were divided into two groups: crude CFS and adjusted CFS. Crude CFSs had a pH of 4.2, while the adjusted CFSs was modified to pH 6.50 using 5 M NaOH (Labscan, Dublin, Ireland). The CFSs were then sterilized using a 0.20 μm syringe filter membrane (Sartorius Stedim Biotech GmbH, Göttingen, Germany).

#### 3.1.3. Antibacterial Activity Using the Agar Well Diffusion Assay

The antibacterial activity of CFSs against clinical strains was evaluated using the agar-well diffusion technique. Cultures of the clinical strains grown overnight were standardized to a 0.5 McFarland standard and spread onto Mueller Hinton agar (MHA) plates (Himedia, Mumbai, India). Wells with a diameter of 9 mm were created in the agar, into which 100 μL of each CFS sample was added. Subsequently, the plates were incubated at 37 °C for 24 h, and the zones of inhibition were measured using vernier caliper. This experimental procedure was conducted in triplicate [46].

### 3.2. Genomic Characterization and Bioinformatics Analysis

#### 3.2.1. Genomic DNA Extraction and Whole-Genome Sequencing

Genomic DNA (gDNA) from ENM104 was isolated using the ZymoBIOMICS DNA Miniprep Kit (Zymo Research, Irvine, CA, USA) following the manufacturer’s guidelines. The concentration and purity of the extracted gDNA were assessed with a Qubit Fluorometer (Invitrogen, Carlsbad, CA, USA), and DNA integrity was confirmed using agarose gel electrophoresis. For whole-genome sequencing (WGS), the purified gDNA was prepared for both short-read and long-read sequencing platforms. Short-read WGS utilized the MGISEQ-2000 platform with 150-bp paired-end reads, while long-read WGS was conducted using the Oxford Nanopore Technologies (ONT) system. The genomic DNA (gDNA) library was created using a Rapid Barcoding Kit 24 V14 (SQK-RAK114.24, Oxford Nanopore Technologies, Oxford, UK) in accordance with the manufacturer’s instructions. Subsequently, the prepared gDNA library was loaded onto the R.10 flow cell and sequenced using a MinION Mk1C sequencer (Oxford Nanopore Technologies) following standard ONT protocols.

#### 3.2.2. Genome Assembly, Annotation, and Visualization

Unicycler version 0.5.0 [47] was utilized for de novo assembly, while Prokka version 1.12 [48] was employed for genome annotation. Functional annotation for ENM104 was conducted using Rapid Annotations using Subsystems Technology (RAST) [49].

#### 3.2.3. In Silico Analysis of Probiotic Properties of *P. pentosaceus* ENM104

A probiotic gene database was created by compiling genes from the literature that are associated with the genus *Pediococcus*. These genes are involved in cell adhesion, stress resistance, vitamin biosynthesis, immunomodulation, DNA and protein protection and repair, and secondary metabolite biosynthesis [50,51]. To find similar genes in our target genome, the Basic Local Alignment Search Tool (BLAST)was used to compare protein sequences (https://blast.ncbi.nlm.nih.gov/Blast.cgi, accessed on 3 February 2024). A cutoff value of 1E-20 and a minimum of 80% identity were applied to ensure that our results are both accurate and reliable. The PathogenFinder (https://cge.food.dtu.dk/services/PathogenFinder/, accessed on 3 February 2024) and Comprehensive Antibiotic Resistance Database (CARD) (https://card.mcmaster.ca/home, accessed on 3 February 2024) were applied for the identification of antibiotic resistance and virulence factor-related genes.

#### 3.2.4. Bacteriocin Identification and Secondary Metabolite Identification

The identification of bacteriocin-encoding genes in the ENM104 was performed using BAGEL 4 [52]. Subsequently, each open reading frame (ORF) in the structure of the detected bacteriocin was verified using BLASTp (https://blast.ncbi.nlm.nih.gov/Blast.cgi, accessed on 3 February 2024). To identify the secondary metabolite encoding genes in the ENM104, antiSMASH version 7.0 was used to predict the ORF and annotate functional characteristics [53].

#### 3.2.5. Cholesterol-Reducing Gene and γ-Aminobutyric Acid (GABA) Synthesis Gene Identification

Genes involved in GABA production pathways and cholesterol reduction were identified using the Kyoto Encyclopedia of Genes and Genomes (KEGG) database (https://www.genome.jp/kegg/pathway, accessed on 3 February 2024). These predictions helped to map the functional roles of the identified genes within the metabolic pathways of *P. pentosaceus* ENM104.

#### 3.2.6. Pan-Genome Analysis of *Pediococcus* Species

The 135 deposited genome assemblies of *P. pentosaceus* strains were downloaded from the National Center for Biotechnology Information (NCBI) database (https://www.ncbi.nlm.nih.gov/, accessed on 3 February 2024) for pan-genome analysis. Annotations for all genomes were created using The General Feature Format (GFF) files generated by Prokka that were analyzed for pan-genome content using Roary version 3.13.0 [54] applying a minimum BLASTP identity of 95% and a core genome threshold of 99%. Subsequently, the matrix, frequency, and pie chart of the genes were visualized using roary_plots.py. The pan-genome profile was analyzed by identifying shared and unique gene clusters among all strains.

#### 3.2.7. Comparative Genomic Analysis of ENM104

Genome sequences of *P. pentosaceus* ENM104 strain and other closely related genomes of *P. pentosaceus* strains MR001 [55] and CGMCC 7049 [56] from the NCBI database were compared and visualized using VENN diagram.

### 3.3. Statistical Analysis

Statistical analysis was conducted using SPSS version 26.0 for Windows (SPSS Inc., Chicago, IL, USA). Results were expressed as means ± SD. One-way analysis of variance (ANOVA) with Tukey’s HSD test was used to compare groups. Differences were considered statistically significant when *p* < 0.05.

## 4. Conclusions

This study unveils the genomic profile of *P. pentosaceus* ENM104, revealing numerous genes linked to probiotic attributes, including stress resistance, vitamin biosynthesis, and immune modulation. Notably, the genome also contains genes responsible for GABA production, cholesterol reduction, secondary metabolite synthesis, and bacteriocin production. Essentially, the genome contains genes encoding for choloylglycine hydrolase, which plays a vital role in cholesterol degradation, and *gadC*, which is involved in GABA transportation.

In addition, the ENM104 strain contains bacteriocin (penocin_A) production genes, as confirmed by in-silico analysis corresponding to previous in vitro results. Importantly, no antibiotic resistance genes or virulence factors were found in either the genome or plasmid of ENM104, underscoring its safety as a probiotic. Comparative genomic analysis identified several distinct genes in ENM104 that contribute to its unique molecular functions and biological processes compared to other strains.

These findings emphasize the potential of *P. pentosaceus* ENM104 as a promising probiotic candidate, with pharmaceutical applications. Future research will focus on exploring the functional aspects of these distinctive genes and their potential applications, including further in vivo studies and clinical trials, to fully understand and harness the benefits of ENM104.

## Figures and Tables

**Table 1:** Inhibition zones of the antibacterial activity of *P. pentosaceus* ENM104 CFS.

Clinical Strains	Inhibition Zones (mm)							
Time (h)	Non-Adjusted				Adjusted			
	12	24	36	48	12	24	36	48
*Pseudomonas aeruginosa* PA01	15.17 ± 0.34 *	15.33 ± 1.25 *	15.67 ± 0.47 *	16.83 ± 0.29 *	N	N	N	N
*Escherichia coli* CREC003	N	N	N	N	N	N	N	N
*Acinetobacter baumannii* CRAB-SK005	12.33 ± 0.47 *	13.33 ± 0.47 *	14.83 ± 0.62 *	15.50 ± 0.50 *	N	N	N	N
*Klebsiella pneumoniae* CRKP10	N	N	N	12.17 ± 0.29 *	N	N	N	N

“N” denotes no inhibition. The asterisk (*) indicates significant differences in each different time point group (*p* < 0.05).

**Table 2:** Genomic data of *Pediococcus pentosaceus* ENM104.

Attribute	Chromosome	Plasmid
Genome size (bp)	1,734,928	71,811
G + C content (%)	37.2	38.1
Number of CDS	1689	85
tRNA	55	0
rRNA	15	0
tmRNA	1	0
RAST subsystems	194	5

**Table 3:** List of probiotic marker genes identified in *P. pentosaceus* ENM104.

Gene	Function
**Stress Resistance**	
**Heat stress**	
*htpX*	Protease HtpX
*hrcA*	Heat-inducible transcription repressor HrcA
*hslO*	33 kDa chaperonin
*dnaK*	Chaperone protein DnaK
*dnaJ*	Chaperone protein DnaJ
*ctsR*	Transcriptional regulator CtsR
*grpE*	Protein GrpE
*clp* operon	ATP-dependent Clp protease
*hslV*	ATP-dependent protease subunit HslV
**Acid stress**	
*atp* operon	ATP synthase subunit
*nhaC*	Na(+)/H(+) antiporter NhaC
**Bile tolerance**	
*ppaC*	Manganese-dependent inorganic pyrophosphatase
*cfa*	Cyclopropane-fatty-acyl-phospholipid synthase
**Vitamin biosynthesis**	
*ribU*	Riboflavin transporter FmnP
*ribZ*	Riboflavin transporter RibZ
*ribF*	Bifunctional riboflavin kinase/FMN adenylyltransferase
*folT*	Folate transporter FolT
*btuD*	Vitamin B12 import ATP-binding protein BtuD
*ytrB*	hypothetical protein
*lnrL*	Linearmycin resistance ATP-binding protein LnrL
**Immunomodulation**	
*dltA*	D-alanine-D-alanyl carrier protein ligase
*dltC*	D-alanyl carrier protein
*dltD*	Protein DltD
**Bacteriocin**	
*pedA*	Bacteriocin pediocin PA-1
**Secondary metabolite biosynthesis**	
*mtgA*	Biosynthetic peptidoglycan transglycosylase

**Table 4:** *Pediococcus* strains with GABA-producing ability reported in the literature.

*Pediococcus* Strains	Isolation Sources	GABA Production	Reference
*Pediococcus pentosaceus* ENM104	Fermented pork sausage	4.49 ± 0.03 μg/mL	[12]
*Pediococcus pentosaceus* JC30	Traditional kimchi	3.32 ± 0.04 mg/g	[35]
*Pediococcus acidilactici* LSF1-1	Fermented fish cake (Som fak)	0.80 ± 0.00 g/L	[34]
*Pediococcus pentosaceus* 56	Traditional Mountain Malga (TMM) cheese	9.6 ± 0.9 mg/L	[36]
*Pediococcus pentosaceus* MN12	Fermented fish sauce (mam nem)	16.8 ± 0.00 mM	[37]

**Table 5:** Selected unique genes only found in *P. pentosaceus* ENM104 divided into 2 groups: molecular function and biological process.

Molecular Function	
**Gene**	**Function**
*sphR*	Alkaline phosphatase synthesis transcriptional regulatory protein SphR
*xylQ*	lpha-xylosidase XylQ
*bglC*	Aryl-phospho-beta-D-glucosidase BglC
*adhR*	HTH-type transcriptional regulator AdhR
*cmtB*	Mannitol-specific cryptic phosphotransferase enzyme IIA component
*lmrA*	Multidrug resistance ABC transporter ATP-binding and permease protein
*fryB*	PTS system fructose-like EIIB component 1
*hprS*	Sensor histidine kinase HprS
*licT*	Transcription antiterminator LicT
*btuD*	Vitamin B12 import ATP-binding protein BtuD
*bglA*	6-phospho-beta-glucosidase BglA
*recD2*	ATP-dependent RecD-like DNA helicase
*lacM*	Beta-galactosidase small subunit
*lpdC*	Gallate decarboxylase
*mngB*	Mannosylglycerate hydrolase
*pox5*	Pyruvate oxidase
*tkt*	Transketolase
**Biological process**	
*sphR*	Alkaline phosphatase synthesis transcriptional regulatory protein SphR
*xylQ*	lpha-xylosidase XylQ
*bglC*	Aryl-phospho-beta-D-glucosidase BglC
*adhR*	HTH-type transcriptional regulator AdhR
*cmtB*	Mannitol-specific cryptic phosphotransferase enzyme IIA component
*lmrA*	Multidrug resistance ABC transporter ATP-binding and permease protein
*fryB*	PTS system fructose-like EIIB component 1
*hprS*	Sensor histidine kinase HprS
*licT*	Transcription antiterminator LicT
*btuD*	Vitamin B12 import ATP-binding protein BtuD
*csbX*	Alpha-ketoglutarate permease
*ulaA*	Ascorbate-specific PTS system EIIC component
*gsiB*	Glucose starvation-inducible protein B
*kup*	Low affinity potassium transport system protein kup
*agaC*	N-acetylgalactosamine permease IIC component 1

**Table 6:** Source and antibacterial resistance profile of *P. pentosaceus* ENM104.

Strains	Specimen	Antibacterial Resistance Profiles											References
		β-Lactam Combination Agents		Carbapenems			Lipopeptide	Aminoglycosides			Fluoroquinolones		
		TZP	C/T	IPM	MEM	DOR	CST	AMK	GEN	TOB	CIP	LVX	
*Pseudomonas aeruginosa* PA01	Rectum	S	S	R	R	R	R	S	S	S	S	S	[42]
*Escherichia coli* CREC003	Rectum	-	-	R	R	R	-	S	S	S	R	R	[43]
*Acinetobacter baumannii* CRAB-SK005	Rectum	-	-	R	R	R	-	-	-	-	-	-	[44]
*Klebsiella pneumoniae* CRKP10	Sputum	R	-	R	R	R	I	R	R	R	R	R	[45]

S, susceptible; I, intermediate; R, resistant; TZP, piperacillin-tazobactam; C/T, ceftolozane-tazobactam; CST, colistin; IPM, imipenem; MEM, meropenem; DOR, doripenem; AMK, amikacin; GEN, gentamicin; TOB, tobramycin; CIP, ciprofloxacin; LVX, levofloxacin.

## Data Availability

The assembled genome of *P. pentosaceus* ENM104 analyzed in this study has been deposited in the NCBI GenBank. The relevant data can be accessed under BioProject number PRJNA1033801 and BioSample number SAMN38044901. The GenBank accession numbers for this dataset are CP137759 and CP137760.

## Supplementary Materials

The following supporting information can be downloaded at: https://www.mdpi.com/article/10.3390/antibiotics13090813/s1, Table S1: General overview of the biological subsystem distribution of the genes in the chromosome of *P. pentosaceus* ENM104; Table S2: General overview of the biological subsystem distribution of the genes in the plasmid in *P. pentosaceus* ENM104.
