# Supplementary material for: Genomic Insights into Pediococcus pentosaceus ENM104: A Probiotic with Potential Antimicrobial and Cholesterol-Reducing Properties
Source: Antibiotics (Basel). 2024 Aug 27;13(9):813. doi: 10.3390/antibiotics13090813 (PMC11428213; doi:10.3390/antibiotics13090813)
Supplement: Supplementary file 1 [file antibiotics-13-00813-s001.zip › antibiotics-3171355-supplementary.pdf]

Table S1. General overview of biological subsystem distribution of the genes in the chromosome of *P. pentosaceus* ENM104.

Description	Value	Percent
Dormancy and Sporulation	6	0.86
Motility and Chemotaxis	0	0
Amino Acids and Derivatives	84	11.98
Stress Response	16	2.28
Cofactors, Vitamins, Prosthetic Groups, Pigments	53	7.56
Sulfur Metabolism	3	0.43
Cell Wall and Capsule	33	4.71
Phages, Prophages, Transposable elements, Plasmids	0	0
Nucleosides and Nucleotides	82	11.7
Iron acquisition and metabolism	4	0.57
Miscellaneous	7	1
Fatty Acids, Lipids, and Isoprenoids	21	3
Metabolism of Aromatic Compounds	1	0.14
Nitrogen Metabolism	0	0
Potassium metabolism	5	0.71
RNA Metabolism	35	5
DNA Metabolism	45	6.42
Carbohydrates	118	16.83
Secondary Metabolism	0	0
Virulence, Disease and Defense	24	3.42
Protein Metabolism	122	17.4

Membrane Transport	13	1.85
Respiration	8	1.14
Photosynthesis	0	0
Cell Division and Cell Cycle	5	0.71
Regulation and Cell signaling	12	1.71
Phosphorus Metabolism	4	0.57

Table S2. General overview of biological subsystem distribution of the genes in the plasmid in *P. pentosaceus* ENM104.

**Description**	**Value**	**Percent**
Amino Acids and Derivatives	2	2.35
DNA Metabolism	1	1.18
Virulence, Disease, and Defense	1	1.18
Nucleosides and Nucleotides	3	3.53
